# Supplementary material for: Clinical presentation, etiology, and treatment outcomes of mycetoma: A 25-year retrospective study in Southern Thailand
Source: PLoS Negl Trop Dis. 2026 Jan 21;20(1):e0013818. doi: 10.1371/journal.pntd.0013818 (PMC12851456; doi:10.1371/journal.pntd.0013818)
Supplement: Attachment — Submitted filename: Response to reviewers 28-11-2025 version02.docx [file pntd.0013818.s003.docx]

**Response to Reviewers**
Title: *Clinical Presentation, Etiology, and Treatment Outcomes of Mycetoma: A 25-Year Retrospective Study in Southern Thailand*
Authors: Sorawit Chittrakarn, Siripen Kanchanasuwan, Natthapat Sangkakul, Nonthanat Tongsengkee
Journal: PLOS Neglected Tropical Diseases
Manuscript Number: PNTD-D-25-01783R1

Dear Editor and Reviewers,

We thank you for the careful evaluation of our manuscript and for providing constructive and insightful comments. We have revised the manuscript accordingly, and all modifications are clearly explained in the detailed point-by-point responses below. We believe these revisions have substantially improved the clarity, rigor, and overall quality of the manuscript. We greatly appreciate the opportunity to revise our work.

**Methods**

**Reviewer #1:** The authors report a case series of mycetoma in Thailand. The article is well-written and interesting. The results are relevant for these NTD. The statistical analysis is appropriate for the article.No concerns noted regarding ethical or regulatory requirements.

Thank you for the encouraging feedback.

**Reviewer #2:** The methodology is well described, although it acknowledges the limitations of a retrospective study.

Thank you for the encouraging feedback.

**Reviewer #3**: As the authors mention, this is a pretty small sample size. The authors may choose to keep this article strictly descriptive and remove the statistical comparisons since the confidence intervals were so wide anyways

Thank you for this helpful suggestion. We agree with the reviewer. In accordance with this comment and the feedback from Reviewer #1, we have removed Table 6 and the corresponding statistical comparisons from the Results section, keeping the manuscript strictly descriptive.

**Results**

**Reviewer #1:** Due to the sample size, the analysis and information on table 6 may be omitted. Figure 2 could be deleted.

Thank you for the comment. We agree with the reviewer. Table 6 and Figure 2 have been removed from the manuscript as suggested.

**Reviewer #2:** The presentation of the results could be improved. The tables could be better presented with the column containing the total number of cases in the rightmost column.

We agree with the reviewer. All relevant tables have been reformatted so that the column containing the total number of cases now appears in the rightmost position among the data columns, as recommended.

In Table 1, the rows containing the locations “Thigh”, “Buttock”, “Back”, “Perineum”, and “Abdominal Wall” do not need to be included in the table, since there were no cases of mycetoma of any type in these locations. Perhaps a note could be added to the legend.

In Table 1, rows with zero cases (e.g., thigh, buttock, back, perineum, abdominal wall) have been removed. A note has been added to the legend stating that no cases were identified in these locations.

Similarly, in Table 4, the rows referring to posaconazole and amikacin or gentamicin also do not need to be included in the table; it would suffice to note in the legend that no case used these medications.

In Table 4, rows for posaconazole and aminoglycosides (amikacin, gentamicin) have been removed. A note has been added in the legend indicating that none of these agents were used.

Figure 2, which shows the overlap of diagnostic approaches, was not immediately clear: 21 patients underwent culture, but in the figure, the number 21 also identifies the 21 patients who underwent only histopathology, which can be confusing. Perhaps a stronger colored line could be added around the circles corresponding to the tests with numbers in the legend.

Thank you for pointing this out. We agree that Figure 2 may be confusing. In line with this comment and the suggestion from Reviewer #1, Figure 2 has been removed from the revised manuscript.

**Reviewer #3:** (No Response)

**Conclusions**

**Reviewer #1:** The conclusions and limitations are adequate. The authors also discuss potential ways to improve care.

Thank you for the encouraging feedback.

**Reviewer #2:** The conclusions are based on the data presented and the limitations of the research are described. The authors highlight that the etiological profile of the disease identified in the country, different from other areas, may demand strategic control actions and differentiated therapies with great relevance to public health.

Thank you for the encouraging feedback.

**Reviewer #3:** Lines 337-338: Is it necessary to identify to species level to guide therapy? In most settings, eumycetoma (for example) will be treated with itraconazole, regardless of causative fungus. While species level identication can be helpful, it may not be critical in managing a patient. Rather distinguishing between bacteria and fungi is vital.

Thank you for this important comment. We agree with the reviewer that, in most clinical settings, the key determinant of management is distinguishing between fungal and bacterial etiologies, and that species-level identification is often supportive rather than essential for initiating therapy. We have revised the Discussion to clarify this point and added that further clinical studies are needed to determine whether in vitro species-level differences translate into meaningful differences in treatment outcomes. This clarification has been incorporated into discussion part. (line 346-352)

Lines 360-361: Phialophora jeanselmei is just an older, updated name for Exophiala jeanselmei

We appreciate the comment. The manuscript has been corrected accordingly, and the name has been updated to Exophiala jeanselmei. (line 374)

Lines 383-385: The mycetoma belt incldues parts of central and south America in which other organisms other than M. mycetomatis almost predominate. Perhaps the authors should point out that the epi differs from high incidence areas on the African continent instead.

Thank you for the insightful comment. We agree with the reviewer. The discussion has been revised to clarify that the epidemiology in our region differs mainly from that observed in high-incidence African regions, rather than the entire “mycetoma belt.” (line 398-399)

**Summary and General Comments**

**Reviewer #1:** The manuscript is well-written.
Please include in the abstract the IQR ranges.
Please detail the culture media used in the center.
Please detail if any patients had a combination antibiotic or antifungal treatment.
Table 6 could be deleted.
Figure 2 could be deleted.

Thank you for your constructive comments. We have addressed all points as follows:

- IQR values have been added to the Abstract. (line 22-23, line 32)
- Details of the culture media used for aerobic, anaerobic, and fungal cultures have been incorporated into the Methods section. (line 129-134)
- We have clarified the use of combination antimicrobial therapy, indicating cases that received combined antifungal or antibacterial regimens. (line 269-272, 281-285)
- Table 6 has been removed as recommended.
- Figure 2 has been deleted to improve clarity.

**Reviewer #2:** This is a relevant study that fills a knowledge gap regarding mycetoma in Southeast Asia, focusing on Thailand. Due to the rarity of the disease and its status as a Neglected Tropical Disease, prospective controlled studies are difficult to conduct. Within the methodological possibilities, the authors managed to extract important information, identifying the peculiar profile of the etiological agents in the country, which may contribute to new therapeutic approaches. The authors provided a good analysis of the research limitations. For these reasons, I would strongly recommend publication with minor adjustments to the presentation of the results.

We appreciate the reviewer’s positive assessment and thoughtful feedback. We have addressed the suggested minor adjustments to improve the presentation of the results, including reformatting the tables (moving the Total column, removing rows with zero cases, and adding explanatory notes) and removing Figure 2.

**Reviewer #3:** Thank you for the opportunity to review this retrospective study on mycetoma in Southern Thailand. These data are critical to better understand the epidemiology this NTD in Southeast Asia. Some comments and thoughts below:

- Lines 78-79: While it could be unique regional patterns, it might also be due to gaps in reporting. Consider adding a note about publication bias.

Thank you for the suggestion. We agree with the reviewer. A statement acknowledging potential publication bias and under-reporting in the region has been added in the revised manuscript. (line 79-81)

- Lines 80-83: The authors mention eumycetoma causative agents from the large literature review, but what about actinomycetoma? Consider listing common agents from that review as well

Thank you for the comment. We apologize for the confusion. The ASM 2024 review included 24,000 mycetoma cases but analyzed only eumycetoma pathogens, and therefore did not list actinomycetoma agents. For actinomycetoma, earlier reviews consistently identify *Nocardia asteroides* and Nocardia *brasiliensis* as the main causative organisms. This clarification has been added to munuscript (line 82-88).

- Lines 85: Is identification of causative agent essential? Or just etiology? Eumycetoma is likely to be treated the same regardless of causative fungi and same with actinomycetoma. The most important aspect is differentiating between fungi vs bacteria. Consider clarifying

Thank you for this important comment. We agree that distinguishing between fungal and bacterial etiology is the key determinant of treatment. As highlighted study by van de Sande et al., species-level identification may still have clinical relevance because, in the absence of clinical trials, differences in in vitro susceptibility and animal model data suggest potential variation in treatment response among causative agents. We have added clarification to the manuscript to reflect that species-level data may inform therapy, although robust clinical evidence remains lacking (line 89-94).

We sincerely thank the Editor and Reviewers for their thoughtful feedback and valuable recommendations. These comments have significantly strengthened our manuscript. We hope that the revised version meets the expectations of the journal and is now suitable for publication in PLOS Neglected Tropical Diseases.

If any additional clarification or further revision is required, we would be pleased to address it.

Sincerely,

Nonthanat Tongsengkee, M.D.

Division of Infectious Diseases, Department of Internal Medicine

Faculty of Medicine, Prince of Songkla University, Songkhla, Thailand

Email: [tnonthanat@gmail.com](mailto:tnonthanat@gmail.com)
